# Monte Carlo Simulation for Polychromatic X-Ray Fluorescence Computed Tomography with Sheet-Beam Geometry

**DOI:** 10.1155/2017/7916260

**Published:** 2017-05-08

**Authors:** Shanghai Jiang, Peng He, Luzhen Deng, Mianyi Chen, Biao Wei

**Affiliations:** ^1^Key Lab of Optoelectronic Technology and Systems, Ministry of Education, Chongqing University, Chongqing 400044, China; ^2^Engineering Research Center of Industrial Computed Tomography Nondestructive Testing, Ministry of Education, Chongqing University, Chongqing 400044, China; ^3^Department of Radiation Physics, The University of Texas MD Anderson Cancer Center, Houston, TX 77030, USA

## Abstract

X-ray fluorescence computed tomography (XFCT) based on sheet beam can save a huge amount of time to obtain a whole set of projections using synchrotron. However, it is clearly unpractical for most biomedical research laboratories. In this paper, polychromatic X-ray fluorescence computed tomography with sheet-beam geometry is tested by Monte Carlo simulation. First, two phantoms (*A* and *B*) filled with PMMA are used to simulate imaging process through GEANT 4. Phantom *A* contains several GNP-loaded regions with the same size (10 mm) in height and diameter but different Au weight concentration ranging from 0.3% to 1.8%. Phantom *B* contains twelve GNP-loaded regions with the same Au weight concentration (1.6%) but different diameter ranging from 1 mm to 9 mm. Second, discretized presentation of imaging model is established to reconstruct more accurate XFCT images. Third, XFCT images of phantoms *A* and *B* are reconstructed by filter back-projection (FBP) and maximum likelihood expectation maximization (MLEM) with and without correction, respectively. Contrast-to-noise ratio (CNR) is calculated to evaluate all the reconstructed images. Our results show that it is feasible for sheet-beam XFCT system based on polychromatic X-ray source and the discretized imaging model can be used to reconstruct more accurate images.

## 1. Introduction

As a promising imaging modality, X-ray computed tomography combining X-ray analysis and tomographic reconstruction algorithm has attracted wide concern in recent years. It can not only measure the distribution of elements but also the content of elements within samples in a nondestructive and noninvasive manner [1–3]. Conventional XFCT techniques with synchrotron source scan samples using the translation-rotation method, which is obviously unsuitable for most biomedical research laboratories due to its huge and expensive equipment.

Some improvements were proposed by other researchers. The synchrotron source is replaced by X-ray tube, and simulated and experimental demonstration of polychromatic-source XFCT were implemented to reduce dose and scan time, which makes benchtop system feasible [4–6]. Although XFCT based on sheet-beam geometry using synchrotron were also developed, fewer researches were done with polychromatic X-ray source [7].

As a contrast agent, gold nanoparticles (GNPs) have attracted wide concern due to their application in cancer detection and therapy [8–10]. Au within samples exposed by X-ray beam mainly emits K-shell X-rays fluorescence, which can be detected to reconstruct XFCT images. In this study, sheet-beam XFCT system based on polychromatic X-ray source was verified by Monte Carlo simulation. First, two phantoms which contained several GNP-loaded regions were imaged through GEANT 4. The XFCT images were reconstructed by FBP and MLEM algorithms. Second, discretized presentation of sheet-beam geometry was established to reconstruct more accurate XFCT images. Contrast-to-noise ratio (CNR) was used to evaluate images quality. At last, CNR for the reconstructed images as functions of Au weight concentration and size of GNP-loaded regions was discussed.

## 2. Principles and Methods

Principles and simulations of polychromatic X-ray fluorescence computed tomography with sheet-beam geometry are presented in this paper. The data analysis techniques and image reconstruction algorithm are described in Sections 2.1–2.4.

### 2.1. Image System

**Figure 1:**
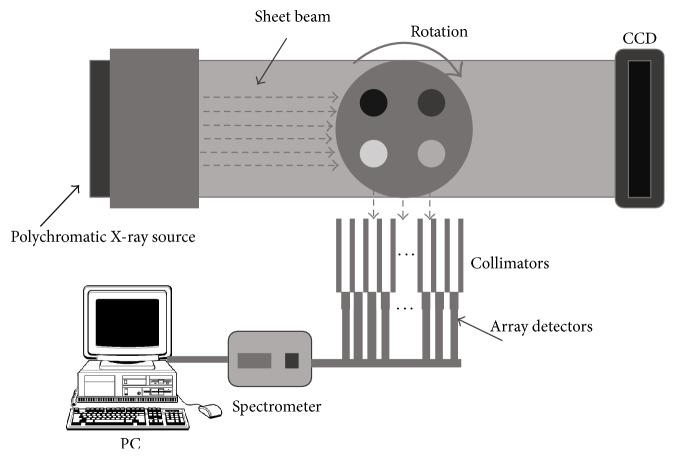
Schematic diagram of XFCT imaging system using sheet-beam and linear detector arrays.

The schematic diagram of sheet-beam CT system proposed in our study is shown in Figure 1. The system includes polychromatic sheet-beam X-ray source, parallel collimator, array detectors, spectrometer, and computer.

Polychromatic X-ray from X-ray tube is collimated into sheet beam and then impinges on the object to cover the whole cross-section. GNPs exposed by X-ray beam can isotropically emit characteristic X-ray photons. Linear array photon-counting detectors with energy resolution are positioned perpendicular to the beam propagation direction for X-ray fluorescent spectra [11].

#### 2.1.1. Monte Carlo Model

**Figure 2:**
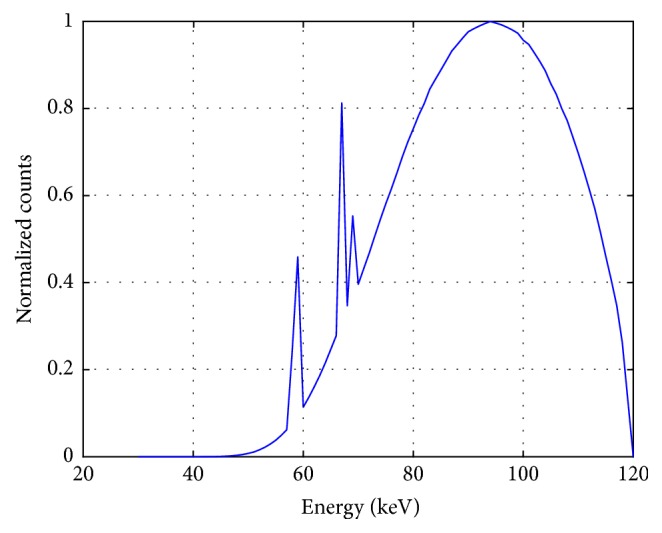
Spectrum of incident polychromatic X-ray source.

The Monte Carlo simulation was completed by GEANT 4 software and ROOT software [12]. Because simulations of projection at each angle are independent of each other, we replace the X-ray source in Figure 1 with a virtual source to reduce simulation time of GEANT 4. The spectrum of sheet-beam X-ray source was calculated by SpekCalc software, which simulates X-ray spectra emitted from thick-target tungsten anode X-ray tubes [10, 13]. In this study, electron beam of 120 keV interacted with tungsten. Then, the emitted X-ray photons were filtered by Sn with thickness of 1 mm. The spectrum of X-ray source was shown in Figure 2. Here, the width and thickness of sheet beam were set to 6.4 cm and 1 mm, respectively.

**Figure 3:**
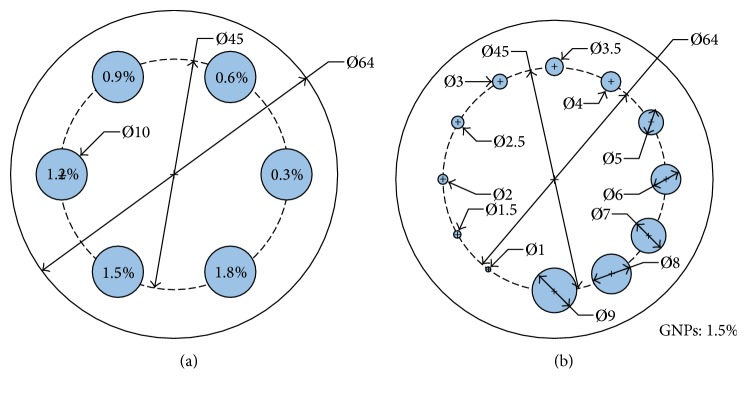
Phantoms contained GNP-loaded regions. (a) GNP-loaded regions with same size in height and diameter but different Au weight concentrations; (b) GNP-loaded regions with same Au weight concentration but different diameters.

Two GNP-loaded PMMA phantoms are shown in Figure 3. The PMMA phantom is 6.4 cm in both height and diameter. The phantom on the left side contained several GNP-loaded regions, which has the same size (10 mm) in height and diameter but different gold concentration (mixed with water) ranging from 0.3% to 1.8%. The GNP-loaded regions in right phantom have the same concentration (1.5%) but different diameter ranging from 1 mm to 9 mm.

The emitted fluorescence photons were detected by a series of energy-sensitive tallies (shown in Figure 1). They were positioned 1 mm behind lead collimator with a series of pinhole openings of diameter 0.5 mm. The whole XFCT scanning procedure was divided into independent simulation for each projection angle. The fluorescent detector array includes 64 energy-sensitive detectors, where each detector has the same sensitive area (0.5 mm × 0.5 mm) and energy resolution (0.5 keV) [14]. When 10 M histories (photons) were calculated for each simulation, the uncertainty was less than 5% for relevant photon energies (50–75 keV).

#### 2.1.2. Data Acquisition

The X-ray photons arriving at the detectors mainly come from both Compton scatter and characteristic X-ray photons. Considering fluorescent field and the attenuation of low-energy photon in the phantom, the gold K_*α*_ lines (67.0 and 68.8 keV) are the best candidates to reconstruct XFCT images in our simulations. To extract fluorescent signal count, cubic polynomial was used to fit the points around the gold fluorescent peaks. The fluorescence signal counts measured of each projection during the simulation were the difference between the measured signal counts and the fitted counts [10, 15]. A sinogram of the gold fluorescence signal counts was reconstructed using the extracted gold fluorescence signal from each projected simulation.

### 2.2. The Imaging Model of Sheet-Beam XFCT

**Figure 4:**
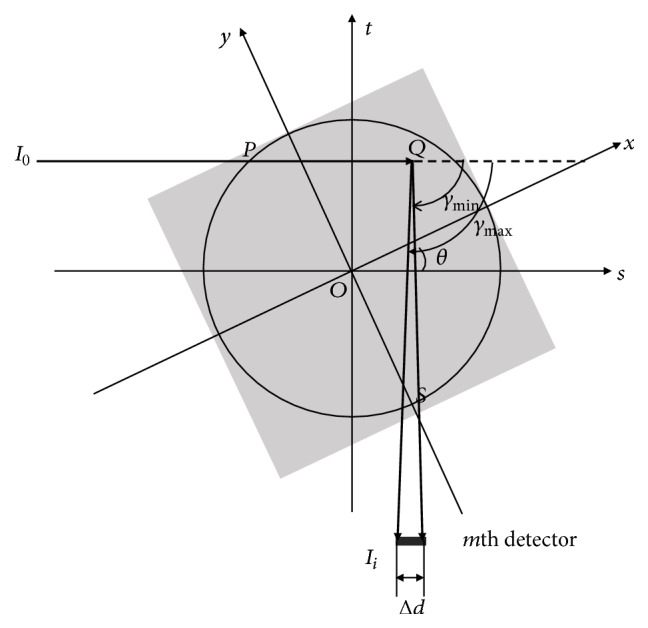
Schematic diagram of XFCT imaging geometry using sheet beam.

The imaging model of sheet-beam XFCT was established previously by some researches [7, 11], and its geometry is presented in Figure 4. While the* xy*-coordinate system is attached to an object, the* st-*coordinate system is spun with the data acquisition system and can be at any instant obtained by rotating the* xy*-coordinate system by an angle *θ* counterclockwise [16]. That is, their relationship can be expressed as follows:

(1) $$\begin{array}{c} \left(\begin{array}{c} s\\ t \end{array}\right)=\left(\begin{array}{cc} \cos\theta & \sin\theta \\ -\sin\theta & \cos\theta \end{array}\right)\left(\begin{array}{c} x\\ y \end{array}\right). \end{array}$$

According to the results of previous research [11, 17–19], the total photons of fluorescent X-ray reaching the *i*th detector are represented as follows:

(2) Iivθ,s=∫−∞+∞fα,s,t•gα,s,t•Ωs,tρx,ydt,

where

(3) fθ,s,t=I0 exp−∫−∞tμIx,yds

(4) gθ,s,t=μphω∫γminγmaxexp⁡−∫0∞μFx,ydbdγ.

*ω* is the yield of characteristic X-ray photons. *Ω* is the solid angle at which the point *Q* is viewed by the *m*th fluorescence detector. *μ*_*ph*_ is the photoelectric linear attenuation coefficient of Au. The *ρ*(*x*, *y*), *μ*^*I*^(*x*, *y*), *μ*^*F*^(*x*, *y*) are the distribution of Au weight concentration, linear attenuation coefficient of incident X-ray energy, and linear coefficient of fluorescent X-ray. Here, in order to simplify reconstruction, (4) can be expressed approximately as follows:

(5) $$\begin{array}{c} I_{iv}\left(\;\theta ,s\;\right)\approx \mu_{ph}\omega I_{0}\Omega \overset{+\infty }{\underset{-\infty }{\int }}\rho \left(\;x,y\;\right)dt. \end{array}$$

Thus, the measured process by the XFCT based on sheet-beam geometry can be viewed approximately as Radon transform, and FBP algorithm can be used to reconstruct XFCT images. Here, the reconstructed images are usually considered to be uncorrected.

**Figure 5:**
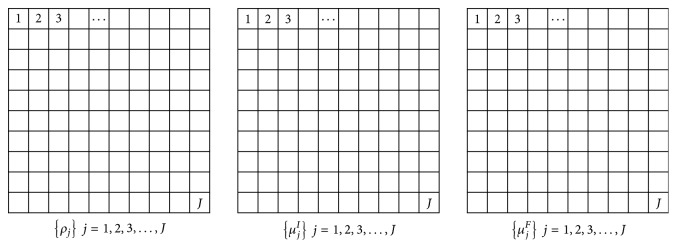
The matrices defined for XFCT.

**Figure 6:**
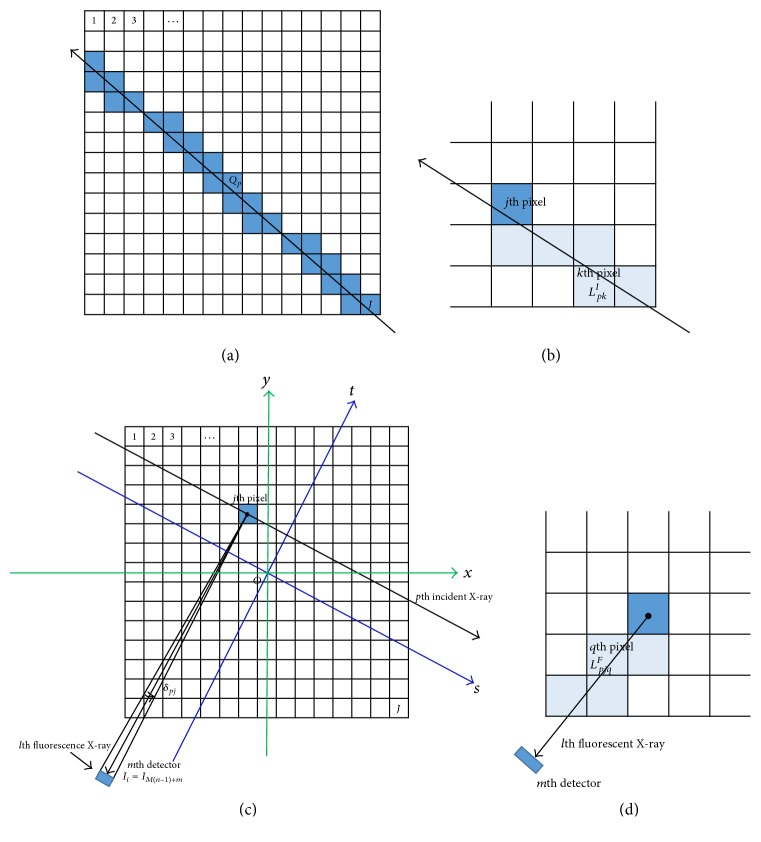
The parameters defined for discretized presentation. (a) A set *Q*_*p*_ defined for blue squares intersected by the* p*th incident X-ray. (b) A set *Q*_*pj*_ defined for light blue squares intersected by the* p*th incident X-ray. (c) Definition of *δ*_*ij*_,* m*. (d) A set* T*_*pjq*_ defined for light blue squares intersected by* l*th fluorescent X-ray.

To acquire the corrected images, it is necessary to obtain discretized representation of (2). During the process, we assume that the phantom is two-dimensional. Three matrices shown in Figure 5, including *ρ*_*j*_, *μ*_*j*_^*I*^, and *μ*_*j*_^*F*^ corresponding to *ρ*(*x*, *y*), *μ*^*I*^(*x*, *y*), and *μ*^*F*^(*x*, *y*), are used to describe the whole phantom, where* j* (*j* = 1,2, 3,…, *J*) represents the number of each pixel. We assume sheet-beam X-ray incident phantom at *N* angles, where *n* (*n* = 1,2, 3,…, *N*) represents the number of each angle. The sheet-beam source is considered as *P* X-rays at each incident direction shown in Figure 6(a). Here, *p*′ is the number of the *p*′th X-ray. We consider the process of the *p*th incident X-ray interacted with phantom. Therefore,

(6) $$\begin{array}{c} p=P\left(\;n-1\;\right)+p^{\prime }. \end{array}$$

**Step 1 .**

Let *Q*_*pj*_ be the subset of *Q*_*p*_, which consists of the light blue pixels shown in Figure 6(b). *L*_*pj*_ is the intersected length of the *p*th X-ray and the *j*th pixel. The incident X-ray intensity before reaching the *j*th pixel is expressed as follows:

(7) $$\begin{array}{c} f_{pj}=I_{0}\exp\left(\;-\underset{k\in Q_{pj}}{\sum }\mu_{k}^{I}L_{pk}^{I}\;\right). \end{array}$$

**Step 2 .**

The total X-ray counts emitted from the *j*th pixel are proportional to the product of *ρ*_*j*_ and *ωμ*_*ph*_*f*_*pj*_*L*_*pj*_^*I*^. Let *δ*_*ij*_ be the angle viewed by the detector corresponding to *i*th projection at the* j*th pixel. Here, we assume that the X-ray counts emitted from the *j*th pixel can be recorded by the *m*th detector, where number *i* can be calculated by (6). The X-ray counts measured by the detector are written as follows:

(8) $$\begin{array}{c} \mu_{ph}\omega \delta_{ij}f_{pj}\rho_{j}L_{pj}^{I}. \end{array}$$

**Step 3 .**

Not all X-ray fluorescence photons emitted from* j*th pixel can be detected by detectors. Here, we assume that the X-ray photos by the* j*th pixel can be detected within *δ*_*ij*_ when the line passing through the center of the* j*th pixel and paralleling to the lead hole can reach detector without block. Attenuation of fluorescent X-ray from* j*th to detector must be also considered during the further process. In Figure 6(c), the fan-shaped X-ray can be divided into* K* (*K* is positive integer) individual X-rays. Let Δ*δ* = *δ*_*pj*_/*K* and* l* (1 ≤ *l* ≤ *K*) is the number of individual fluorescent X-rays. The attenuation of the* l*th fluorescent X-ray can be expressed as follows:

(9) $$\begin{array}{c} \exp\left(\;-\underset{q\in T_{pjl}}{\sum }\mu_{q}^{F}L_{pjq}^{F}\;\right), \end{array}$$

where* T*_*pjl*_ is defined as the set consisting of the pixels (light blue squares shown in Figure 6(d)) intersected with the* l*th fluorescent X-ray. *L*_*pjq*_^*F*^ is described as the intersected length of the* l*th fluorescent X-ray with the *q*th pixel (*q* ∈ *T*_*pjl*_).

(10) $$\begin{array}{c} g_{pij}=\mu_{ph}\omega \delta_{pj}\overset{K}{\underset{l=1}{\sum }}exp\left(\;-\underset{q\in T_{pjl}}{\sum }\mu_{q}^{F}L_{pjq}^{F}\;\right). \end{array}$$

**Step 4 .**

We consider the process of the* p*th incident X-ray interacted with phantom. Let us define that *i* is the number of *i*th projection ranging from 1 to *I*. The contribution *h*_*ij*_ of the* j*th pixel to the *i*th projection at* p*th incident X-ray can be expressed as follows:

(11) i=Mn−1+mhij=fpjgpijδij.

Accordingly, the discretized representation of (4) is written as follows:

(12) $$\begin{array}{c} I_{i}=\underset{j}{\sum }h_{ij}\rho_{j}\;\left(i=1,2,\ldots ,I\right). \end{array}$$

The matrix representation of (9) is

(13) $$\begin{array}{c} I=H\rho , \end{array}$$

where

(14) H=hij1≤i≤M,1≤j≤NI=Ii1≤i≤M,ρ=ρj1≤j≤N.

### 2.3. XFCT Image Reconstruction

Here, we assume that the maps of *μ*^*I*^ and *μ*^*F*^ are known in our study. Two algorithms, including FBP and MELM, were used to reconstruct XFCT images with correction and without correction, respectively. First, sinograms of two phantoms with GNP-loaded regions were acquired by the described method in Section 2.1.2. Then, XFCT images with 64 × 64 pixels of 64 × 64 mm^2^ were reconstructed by FBP and MLEM without correction. The more accurate projection matrix described in (6)–(15) was calculated to correct the effect of attenuation for high quality images.

### 2.4. XFCT Image Analysis

The reconstructed XFCT images are evaluated as CNR by calculating the ratio of difference between the mean value of each GNP-loaded region and background (PMMA) and standard deviation of background. CNR is defined as follows [16, 20]:

(15) $$\begin{array}{c} CNR=\frac{{\overset{-}{\Psi }}_{Region}-{\overset{-}{\Psi }}_{BK}}{V_{BK}}, \end{array}$$

where ${\overset{-}{\Psi }}_{Region}$ and ${\overset{-}{\Psi }}_{BK}$ are mean reconstructed values of GNP-loaded region and background and *V*_*BK*_ is standard deviation of background (PMMA). According to the Rose criterion, imaging sensitivity limit of the system proposed was determined using CNR of 4 [21].

## 3. Results

### 3.1. XFCT Image Reconstruction

**Figure 7:**
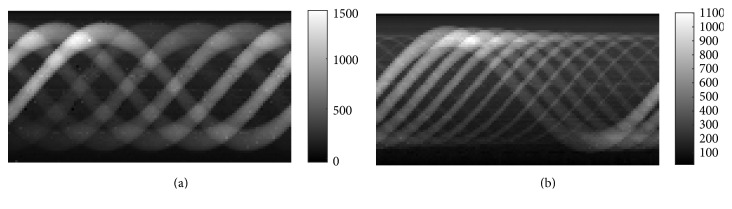
The reconstructed sinograms of (a) phantom *A* and (b) phantom *B*.

**Figure 8:**
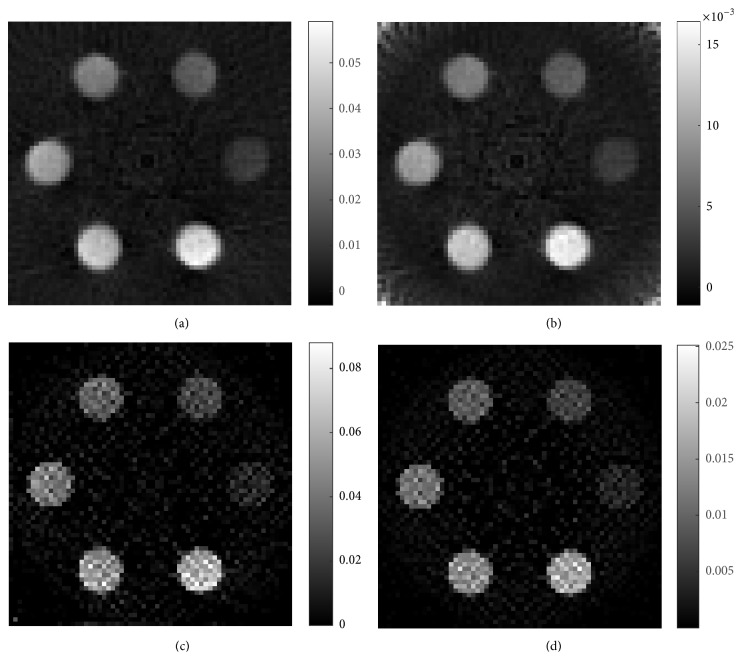
Reconstructed XFCT images of phantom *A*. (a) Reconstructed by FBP without correction, (b) reconstructed by FBP with correction, (c) reconstructed by MLEM without correction, and (d) reconstructed by MLEM with correction.

**Figure 9:**
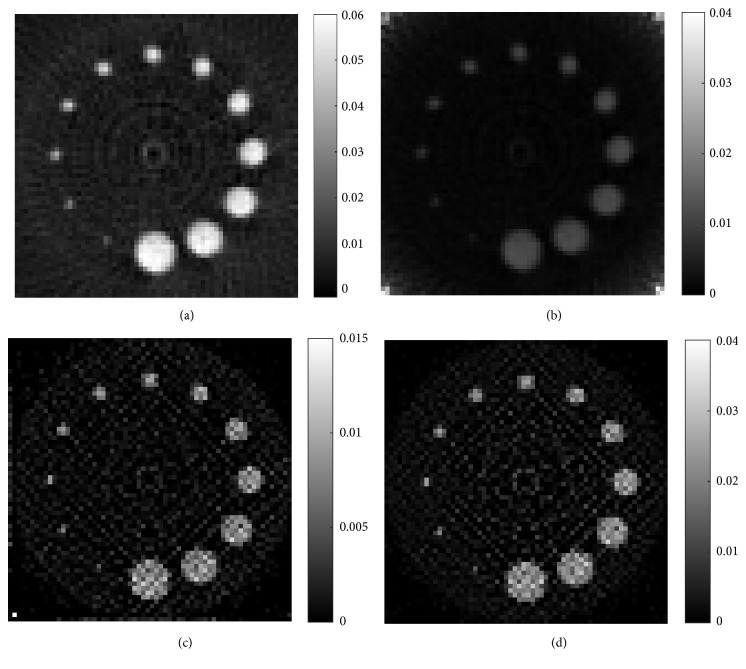
Reconstructed XFCT images of phantom *B*. (a) Reconstructed by FBP without correction, (b) reconstructed by FBP with correction, (c) reconstructed by MLEM without correction, and (d) reconstructed by MLEM with correction.

Sinograms of two phantoms with GNP-loaded regions are shown in Figure 7.  Figure 7(a) shows the sinogram of phantom *A* and Figure 7(b) is the sinogram of phantom *B*. The reconstructed XFCT images of phantom *A* are shown in Figure 8, where Figures 8(a) and 8(c) are the images reconstructed by FBP and MLEM (100 iterations) without correction, respectively. Correspondingly, Figures 9(b) and 9(d) are reconstructed by FBP and MLEM (100 iterations) with correction, respectively. Similar representation is also shown in Figure 9. Obviously, grey values of GNP-loaded regions decrease with reduction of Au weight concentration shown in Figure 8.

**Figure 10:**
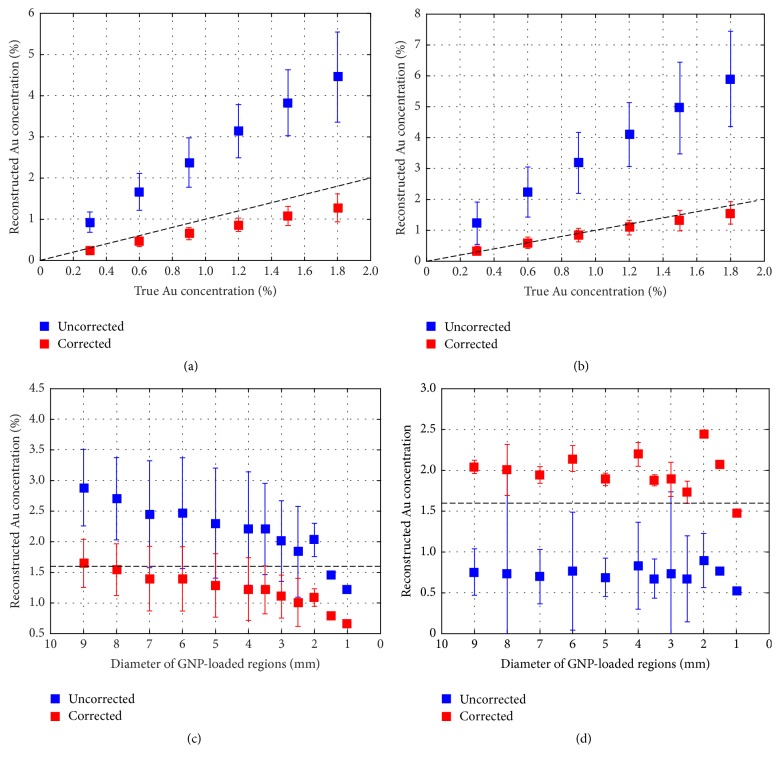
Reconstructed Au weight concentration. (a) and (c) Acquired by FBP algorithm with and without correction. (b) and (d) Acquired by MLEM algorithm with and without correction.

The reconstructed Au concentration calculated from the mean value of each GNP-loaded region in phantom *A* is plotted in Figure 10(a) (acquired by FBP) and Figure 10(b) (acquired by MLEM). Both figures show that uncorrected Au weight concentration has larger error than corrected concentration, which may mean that our corrected model can provide more accurate results. For the same Au weight concentration, the reconstructed values in Figure 10(d) acquired by MLEM algorithm have more stable values than in Figure 10(c) acquired by FBP algorithm.

### 3.2. XFCT Image Analysis

**Figure 11:**
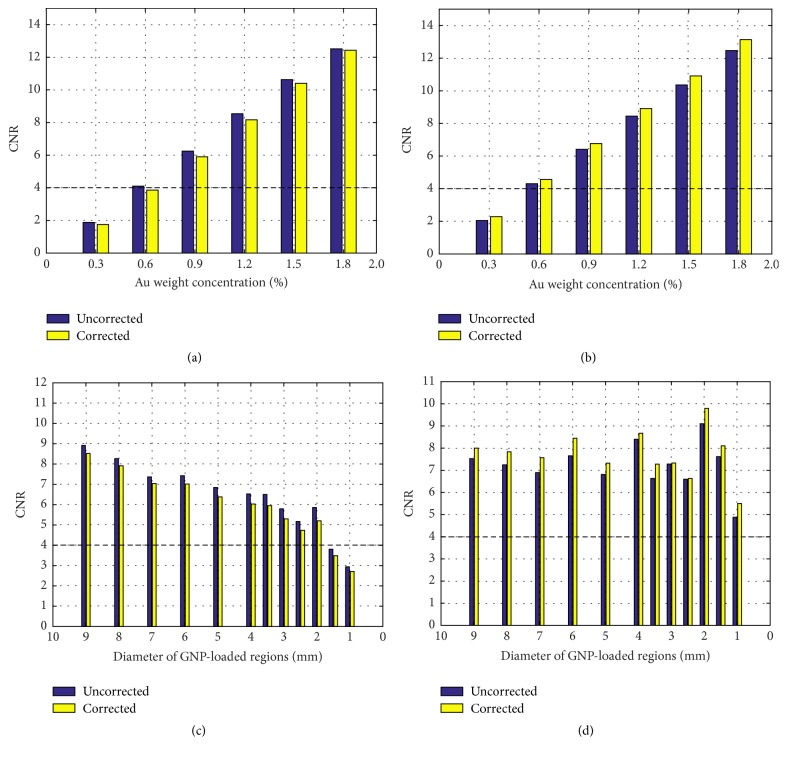
CNR. (a) and (c) Acquired by FBP algorithm with and without correction. (b) and (d) Acquired by MLEM algorithm with and without correction.

CNR for reconstructed XFCT images with FBP and MLEM as a function of Au weight concentration are presented in Figures 11(a) and 11(b), respectively. Both of bar charts show us that values of CNR in our setup have higher than 4, when Au weight concentration is greater than 0.6%. To detect lower concentration, the setup needs to be modified, including length and diameter of collimators, spectrum of X-ray source, and distance from X-ray source to the center of phantom [22]. Figures 11(a) and 11(b) can also indicate that CNR of GNP-loaded region for same size is linearly proportional to Au weight concentration (*R*^2^ ≥ 0.9992). According to Rose criterion (CNR > 4), not all GNP-loaded regions in Figure 8 were detectable, and detection limits from Figures 8(a)to 8(d) were 0.59%, 0.62%, 0.60%, and 0.56%, respectively.

CNR for reconstructed XFCT images with FBP and MLEM as a function of each GNP-loaded region size are also presented in Figures 11(c) and 11(d), respectively. When the diameter of GNP-loaded region is ranging from 7 mm to 9 mm, the values of CNR increase with increase of diameter but fluctuate largely from 1 mm to 6 mm. The phenomenon illustrates that size of GNP-loaded region can influence values of CNR when incident X-ray width is invariant.

Algorithms can influence values of CNR. In Figures 11(a) and 11(c), the corrected images have lower CNR than uncorrected ones with FBP. On the contrary, the corrected images with MLEM have greater than the uncorrected ones in Figures 11(b) and 11(d). However, the corrected concentrations have more accurate than the uncorrected ones.

## 4. Discussion

We have presented a benchtop system for polychromatic X-ray fluorescence computed tomography with sheet-beam geometry through Monte Carlo simulation. The discretized model with sheet-beam XFCT is also described in our study.

In the simulation, we used polychromatic X-rays (X-ray tubes) instead of synchrotron radiation in similar imaging system proposed previously by others [7], which make it possible to reduce costs and size of apparatus. Another advantage of XFCT imaging with sheet-beam geometry is a drastic reduction of overall scanning time, compared to traditional XFCT [10]. Although the proposed XFCT system may not be demonstrated by experimental study, it may provide valuable method for optimization of XFCT system.

However, a technical challenge for the proposed system is improvement of detection limit and CNR. First, they may be improved further by additional modifications to the current setup such as quasi-monochromatization of incident X-ray spectrum and further optimization of detector collimation [7, 19]. Second, the optimized algorithm may improve image quality. According to our results, different algorithms influence the values of CNR, which is similar with conclusion of Di et al. [23]. Thirdly, X-ray detector with higher energy resolution is used during the process. Another potential approach is to consider both *K*_*α*_ peaks and *K*_*β*_ peaks at the same time.

Our future work will consist of optimizing polychromatic XFCT with sheet-beam geometry, such as spectrum of X-ray source and length of collimators. We will also build an imaging system based on our simulations to demonstrate the feasibility by experiment.

## 5. Conclusion 

In this investigation, the feasibility of polychromatic sheet-beam XFCT system proposed in this study was demonstrated by Monte Carlo method. Two phantoms which contained several GNP-loaded regions were imaged using GEANT 4. Accurate images were reconstructed by FBP and MLEM with and without correction, respectively. Our results may provide necessary justification for the design of benchtop XFCT imaging system for in vivo imaging.
